# Association of a Fruit and Vegetable Subsidy Program With Food Purchases by Individuals With Low Income in the US

**DOI:** 10.1001/jamanetworkopen.2021.20377

**Published:** 2021-08-11

**Authors:** Seth A. Berkowitz, Neal Curran, Sam Hoeffler, Richard Henderson, Ashley Price, Shu Wen Ng

**Affiliations:** 1 Division of General Medicine and Clinical Epidemiology, Department of Medicine, University of North Carolina at Chapel Hill School of Medicine; 2 Center for Health Equity Research, Department of Social Medicine, University of North Carolina at Chapel Hill School of Medicine; 3 Reinvestment Partners, Durham, North Carolina; 4 Carolina Population Center, University of North Carolina at Chapel Hill; 5 Department of Family Medicine and Community Health, Duke University School of Medicine, Durham, North Carolina; 6 Department of Nutrition, Gillings School of Global Public Health, University of North Carolina at Chapel Hill

## Abstract

**Importance:**

Food insecurity is associated with a less healthy diet. The Supplemental Nutrition Assistance Program (SNAP) is associated with reduced food insecurity, but benefit levels may be insufficient for beneficiaries to afford healthy foods.

**Objective:**

To evaluate whether participation in SuperSNAP, a program that provides an additional $40 per month for the purchase of fruits and vegetables with no added sugar, sodium, or fat to SNAP beneficiaries, is associated with changes in food purchasing.

**Design, Setting, and Participants:**

This longitudinal cohort study used data from transaction records of a large supermarket chain with approximately 500 stores located across North Carolina from October 2019 to April 2020. Participants were SNAP beneficiaries.

**Exposure:**

SuperSNAP participation.

**Main Outcomes and Measures:**

Monthly spending on all fruits, vegetables, legumes, and nuts (primary outcome); spending on less healthy food categories; and spending on sugar-sweetened beverages as 1 category of less healthy foods. Monthly data on purchases by SNAP beneficiaries before and during SuperSNAP participation were compared with data from SNAP beneficiaries not enrolled in the program who shopped at the same stores. Overlap weighting (a propensity score–based method) was used to account for confounding, and linear mixed-effects models were fitted with random effects to account for repeated measures and clustering by store.

**Results:**

The study included 667 SuperSNAP participants and 33 246 SNAP beneficiaries who did not use SuperSNAP but shopped in the same stores; 436 SuperSNAP participants had preintervention data and were included in the main analysis. SuperSNAP participation was associated with increased monthly purchases of fruits, vegetables, nuts, and legumes ($31.84; 95% CI, $31.27-$32.42; *P* < .001; 294.52 oz; 95% CI, 288.84-300.20 oz; *P* < .001). Only a small increase in spending on less healthy food categories compared with the SNAP beneficiaries who did not use SuperSNAP ($1.60; 95% CI, $0.67-$2.53; *P* < .001) was observed. As total spending increased, the proportion of total food and beverage spending on less healthy foods significantly decreased (difference, 4.51%; 95% CI, 4.27%-4.74%; *P* < .001). Monthly spending on sugar-sweetened beverages decreased (difference, $1.83; 95% CI, $1.30-$2.36; *P* < .001).

**Conclusions and Relevance:**

In this cohort study, participation in SuperSNAP was associated with meaningful increases in healthy food purchasing. Subsequent studies should investigate whether healthy food incentive programs improve health outcomes.

## Key Points

**Question:**

Is a program that provides additional funds to Supplemental Nutrition Assistance Program (SNAP) beneficiaries for purchase of fruits and vegetables (SuperSNAP) associated with changes in food purchasing?

**Findings:**

In this cohort study of 667 SuperSNAP participants and 33 246 SNAP beneficiaries not enrolled in SuperSNAP, participation in SuperSNAP was associated with a significant increase in purchases of fruits and vegetables of $31.84 per month.

**Meaning:**

The findings suggest that a program that incentivizes the purchase of healthier foods for SNAP beneficiaries may be associated with improved healthfulness of food purchases.

## Teaser

This cohort study of Supplemental Nutrition Assistance Program (SNAP) beneficiaries evaluates whether participation in SuperSNAP, a program that provides additional funds for purchase of fruits and vegetables, is associated with purchases of healthier food.

## Introduction

Greater fruit and vegetable consumption and less sodium, sugar, and saturated fat consumption are associated with longer life and reduced risk of poor health.^1,2,3,4,5,6,7,8,9,10,11,12,13^ In 2019, more than 10% of US households were food insecure, meaning that they had uncertain access to enough food for an active, healthy life; this percentage has increased during the COVID-19 pandemic.^14,15^ Food insecurity is associated with the consumption of less healthy foods that are cheaper on a per-calorie basis than healthy foods.^16,17,18^ The Supplemental Nutrition Assistance Program (SNAP) is the largest program in the US to address food insecurity.^19^ Although SNAP is associated with reduced food insecurity, current SNAP benefit amounts are often insufficient to allow purchase of the recommended amounts of fruits and vegetables.^20,21,22^

Healthy food incentive programs, which provide additional funds to facilitate the purchase of healthier foods, have emerged as a key intervention to reduce food insecurity and improve diet.^23,24,25,26^ In this study, we evaluated SuperSNAP,^27^ a program funded by a US Department of Agriculture grant (formerly Food Insecurity Nutrition Incentive and now known as the Gus Schumacher Nutrition Incentive Program),^28^ that was designed to improve diet quality for SNAP beneficiaries.

Because the primary mechanism whereby SuperSNAP may improve health is through changes in food purchasing, evaluating whether changes in food purchasing occur would be informative. Proof of this concept would support further investigation into whether programs such as SuperSNAP have effects on diet quality and health. A recent report^29^ identified the following recommendations for food subsidy programs: electronic issuance, longer duration (>24 weeks) with repeated incentives, coverage of a broad selection of healthy foods (eg, including frozen and canned fruits and vegetables rather than only fresh produce), and redemption in stores (vs solely in farmers’ markets). Because SuperSNAP has several of these features, we used this program to evaluate whether providing incentives to purchase healthy foods is associated with changes in food purchasing. We hypothesized that SNAP beneficiaries who participated in SuperSNAP, compared with SNAP beneficiaries who did not participate in the program, would have increased fruit and vegetable purchasing.

## Methods

### Study Design, Setting, and Participants

This retrospective cohort study was performed from October 2019 to April 2020 with a dynamic cohort, meaning that participants could enter and exit the cohort at different times. The primary source of data was the transaction records of a large supermarket chain with approximately 500 stores located across North Carolina. The University of North Carolina institutional review board did not consider this study of deidentified data to be human participant research; therefore, the requirement for informed consent was waived. This study followed the Strengthening the Reporting of Observational Studies in Epidemiology (STROBE) reporting guideline.

The unit of analysis was the monthly transactions linked to a particular shopper identification (ID) number. The shopper ID number is a unique identifier available to all shoppers that can be used to obtain store discounts on purchases. The ID numbers were used to track SuperSNAP benefit use, and all SuperSNAP benefits were linked to a unique shopper ID number. For this study, we included all transaction data in a given month in which a shopper ID number was linked to a purchase that used any SNAP benefits during that month. SNAP benefits only needed to be used for 1 transaction (not every transaction) in a given month to be included in this study.

### SuperSNAP

SuperSNAP provided $40 per month in additional funds to SNAP beneficiaries that could be spent on fresh, frozen, or canned fruits and vegetables with no added sugar, fat, or salt. SuperSNAP benefits could be redeemed at any location of a single supermarket chain. Eligible foods were programmed into the supermarket point-of-sale system, and when eligible foods were purchased, the cost of those foods was deducted from the available SuperSNAP balance at the point of purchase. SuperSNAP participants were adults (age, ≥18 years) who were current SNAP beneficiaries and identified by staff at their primary care clinic as likely to benefit from the program because of their health status (eg, owing to diabetes or obesity). Participants were enrolled by clinic staff at 9 clinics—primarily federally qualified health care centers—throughout North Carolina. Participants completed a brief enrollment survey with clinic staff; the survey included questions about household composition, food security (using the Hunger Vital Sign 2-item screener),^30^ and proportion of food shopping done at the supermarket chain. Access to the program expired if participants did not spend any SuperSNAP benefits for 2 consecutive months, and unused benefits at the end of each month rolled over for 2 months.

### Data Collection and Categorization of Spending

The transaction data included every item bought during each shopping episode and the payment method. Eligible fruit and vegetable purchases made through SuperSNAP were identified based on a unique offer code applied at checkout. We were thus able to determine the source of food spending for each transaction (eg, SNAP, SuperSNAP, or out of pocket). All transactions within a given month were aggregated to produce monthly data for each shopper ID.

On the basis of other ongoing work using large, national data sets on household purchases and nutritional label data at the barcode level,^31,32^ we were also able to categorize every purchased item as a nonfood item (eg, toilet paper, magazine, or tobacco) or a food item. Among food items, we further examined 3 specific categories as being particularly relevant for health outcomes (eTable 1 in the Supplement): (1) fruits, vegetables, legumes, and nuts; (2) less healthy foods including processed meats and processed seafood, desserts, sweet snacks, salty snacks, candy, chocolate, gum, sweeteners, and toppings; and (3) sugar-sweetened beverages.

### Outcomes

The primary outcome for this study was total expenditure, in US dollars, from any spending source on all fruits, vegetables, legumes, and nuts. Secondary outcomes were spending on fruits, vegetables, nuts, and legumes in ounces and as a proportion of total spending on food and beverages and out-of-pocket spending on fruits, vegetables, nuts, and legumes as a proportion of total spending on food and beverages. Because food incentives, even if restricted to healthier foods, may allow for resources to be spent on less healthy foods, thus negating the potential impact of a healthy food incentive, we included these secondary outcomes examining spending on less healthy food categories. Furthermore, because prior research has suggested that consumption of sugar-sweetened beverages, which is associated with poor health, may decrease when healthier foods are subsidized,^25,33^ we examined sugar-sweetened beverages as a subcategory of less healthy foods. In terms of spending categories, we considered total spending (eg, from all sources) and out-of-pocket spending (eg, spending other than SNAP and SuperSNAP benefit use).

### Statistical Analysis

We sought to analyze whether participation in SuperSNAP was associated with changes in food purchasing. We analyzed data on monthly purchases by SNAP beneficiaries before and during SuperSNAP participation compared with data from SNAP beneficiaries who never enrolled in the program and shopped at the same stores (ie, 2 groups were observed during 2 periods). The 2 periods were demarcated by an index date, which divided the study into periods before and after the index date. For those who participated in SuperSNAP, the index date was defined as the month in which SuperSNAP participation began. For the comparison group of SNAP beneficiaries who never enrolled in SuperSNAP, the index date was selected at random from a distribution that matched the distribution of SuperSNAP enrollment months. To help account for confounding, we used a propensity score–based analytic strategy called overlap weighting,^34,35^ which assigns an overlap weight equal to the estimated probability (ie, the propensity score) of being in the group in which the participant did not actually belong. Overlap weighting has the property of exactly balancing all covariates used to estimate the propensity score (when estimated with a logistic regression model).^34^ To calculate the propensity score, we used purchase record data organized by shopper ID number. Only data from before the index date were used to estimate the propensity score. To enhance internal validity, we excluded shopper ID numbers that did not have observations before the index date because we would not be able to estimate a propensity score for those shopper ID numbers. To estimate the propensity score, we fit logistic regression models that estimated the probability of SuperSNAP participation based on the variables listed in the eAppendix in the Supplement, encompassing absolute and relative measures of expenditures across different categories, absolute measures on volume or weight purchased across different categories, relative measures of payment source for different categories, and time measures.

To evaluate whether there were changes in purchasing associated with SuperSNAP participation, we fit linear mixed models for the aforementioned outcomes. The unit of analysis was the shopper-month. The models included terms for SuperSNAP participation (1 or 0), time in relation to index date (0, before index date; 1, on or after index date), and a SuperSNAP × time interaction term to estimate whether a given outcome differed between those who participated in SuperSNAP and those who did not after the index date. To account for seasonality in shopping and secular trends (eg, the COVID-19 pandemic), we adjusted for the month and year of observation and the duration of follow-up time. Because follow-up time was a variable after the index date, it was not balanced by overlap weighting. Because the store at which an individual shopped may have provided information about geographic characteristics and socioeconomic status and because those who shopped at the same stores may have been more similar than those who shopped at different stores, we used a random intercept term for store ID (the store that received a plurality of expenditures for a given shopper ID in a given month). To account for repeated measurements within shopper ID numbers, we also included a random intercept term for shopper ID number.

Although the overlap weighting approach used enhanced internal validity, this approach could also affect generalizability of the findings because SuperSNAP participants without pre-SuperSNAP data were excluded. Therefore, we conducted additional analyses that included all shopper ID numbers for shoppers who used SuperSNAP even if there were no pre-SuperSNAP data. For these analyses, we used the same modeling approach as described above but without weighting.

Analyses were conducted using SAS, version 9.4 (SAS Institute Inc). A 2-sided *P* < .05 was considered to indicate statistical significance. The overlap-weighted analysis with the fruit, vegetable, nut, and legume spending outcome was considered primary, and other analyses were considered secondary.

## Results

Overall, 824 shoppers were enrolled in SuperSNAP from October 2019 to April 2020. Of these, 667 used SuperSNAP benefits (81.0% SuperSNAP redemption rate) at some time during this period, and 436 had preintervention data and were thus included in the main analysis. The comparison group included 33 246 SNAP beneficiaries who never enrolled in SuperSNAP. The 667 shoppers who had used SuperSNAP benefits were included in the sensitivity analyses. Table 1 shows characteristics of those who were enrolled in SuperSNAP. The mean (SD) household size was 2.3 (1.7; range, 1-12), and 341 (91.7%) participants reported having experienced food insecurity. SuperSNAP participants who had pre-SuperSNAP data available were similar to those who did not have these data available.

**Table 1.** Characteristics of SuperSNAP Participants

Characteristic	Participants^a^		
	Enrolled in SuperSNAP (n = 824)	Ever used SuperSNAP (n = 667)	Used SuperSNAP and had preintervention data available (n = 436)^b^
Household size, mean (SD), individuals	2.2 (1.7)	2.2 (1.7)	2.3 (1.7)
Households with at least 1 member aged <18 y	252 (31.1)	205 (31.2)	146 (34.3)
Households with at least 1 member aged ≥65 y	170 (20.9)	135 (20.5)	93 (21.5)
Food insecure	638 (93.0)	505 (92.5)	341 (91.7)
Participate in WIC	122 (16.9)	102 (17.9)	74 (19.0)
Frequency of monthly food shopping at SuperSNAP grocery store^c^			
Never	18 (2.2)	12 (1.8)	2 (0.4)
Seldom	116 (14.1)	86 (12.9)	41 (9.4)
About half the time	184 (22.4)	146 (21.9)	96 (22.0)
Usually	187 (22.7)	157 (23.5)	118 (27.1)
Always	194 (23.6)	155 (23.2)	123 (28.2)

Abbreviations: SNAP, Supplemental Nutrition Assistance Program; WIC, Special Supplemental Nutrition Program for Women, Infants, and Children.

^a^ Cell totals may not equal cohort totals owing to missing survey responses. Data are presented as number (percentage) of participants unless otherwise indicated.

^b^ Main analysis cohort.

^c^ Responses are from the SuperSNAP enrollment survey.

Table 2 shows purchasing characteristics before the index date. Before weighting, mean (SD) monthly total spending on food and beverages was higher in the comparison group than in the SuperSNAP group ($267.01 [$238.15] vs $235.95 [$216.39]; *P* = .02), but mean (SD) monthly spending on fruits, vegetables, nuts, and legumes ($32.97 [$36.25] vs $34.00 [$40.46]; *P* = .68) and mean (SD) monthly spending on sugar-sweetened beverages ($33.59 [$43.16] vs $32.31 [$43.33]; *P* = .63) were similar. In the weighted comparisons, all characteristics were exactly balanced. There was a small difference in the mean (SD) duration of follow-up between SuperSNAP participants and the comparison group (3.0 [1.5] months vs 2.7 [0.1] months; *P* < .001); thus, regression analyses adjusted for duration of follow-up.

**Table 2.** Differences in Preintervention Purchases Between 436 SuperSNAP Participants and 33 246 Comparison Group Participants

Variable	Overall	Unweighted analysis			Weighted analysis^a^	
		Comparison group^b^	SuperSNAP group	*P* value^c^	Comparison group^b^	SuperSNAP group
All grocery store spending, mean (SD), $	341.54 (311.60)	341.81 (311.78)	296.88 (277.09)	.009	296.72 (21.85)	296.72 (275.60)
Food and beverage spending, mean (SD), $	266.83 (238.03)	267.01 (238.15)	235.95 (216.39)	.02	235.73 (17.03)	235.73 (215.04)
Fruits, vegetables, nuts, and legumes spending, mean (SD), $	32.97 (36.28)	32.97 (36.25)	34.00 (40.46)	.68	33.87 (3.11)	33.87 (39.92)
Fruits, vegetables, nuts, and legumes spending, mean (SD), oz	318.84 (352.63)	318.70 (352.32)	341.70 (400.82)	.35	340.46 (31.76)	340.46 (395.15)
Proportion of fruits, vegetables, nuts, and legumes spending among total food and beverage spending, mean (SD)	0.13 (0.11)	0.13 (0.11)	0.15 (0.14)	<.001	0.15 (0.01)	0.15 (0.14)
Proportion of out-of-pocket fruits, vegetables, nuts, and legumes spending among total food and beverage spending, mean (SD)	0.41 (0.35)	0.41 (0.35)	0.38 (0.35)	.16	0.38 (0.03)	0.38 (0.34)
Spending on less healthy food categories, $	77.58 (75.89)	77.65 (75.94)	64.74 (65.96)	<.001	64.76 (5.25)	64.76 (65.67)
Spending on less healthy food categories, mean (SD), oz	381.99 (389.25)	382.18 (389.31)	350.26 (377.72)	.14	350.17 (31.80)	350.17 (375.77)
Proportion of spending on less healthy food categories among total food and beverage spending, mean (SD)	0.30 (0.15)	0.30 (0.15)	0.28 (0.16)	.03	0.28 (0.01)	0.28 (0.15)
Proportion of out-of-pocket spending on less healthy food categories among total spending on less healthy food categories, mean (SD)	0.42 (0.32)	0.42 (0.32)	0.39 (0.32)	.03	0.39 (0.03)	0.39 (0.32)
Sugar-sweetened beverage spending, $	33.58 (43.16)	33.59 (43.16)	32.31 (43.33)	.63	32.27 (3.70)	32.27 (43.05)
Sugar-sweetened beverage spending, mean (SD), oz	915.29 (1153.14)	915.23 (1152.63)	926.29 (1235.54)	.88	924.54 (115.28)	924.54 (1226.06)
Proportion of sugar-sweetened beverage spending among total food and beverage spending, mean (SD)	0.14 (0.16)	0.14 (0.16)	0.14 (0.16)	.35	0.14 (0.01)	0.14 (0.16)
Proportion of out-of-pocket, sugar-sweetened beverage spending among total sugar-sweetened beverage spending, mean (SD)	0.40 (0.36)	0.40 (0.36)	0.36 (0.36)	.03	0.36 (0.03)	0.36 (0.36)

Abbreviation: SNAP, Supplemental Nutrition Assistance Program.

^a^ Overlap weighting was used to assign an overlap weight equal to the estimated probability (ie, the propensity score) of being in the group in which the participant did not actually belong. Because overlap weights produce exactly balanced means, we did not calculate *P* values comparing means in the weighted sample.

^b^ Comparison group participants were SNAP beneficiaries who shopped in the same stores during the same months as SuperSNAP participants but did not participate in SuperSNAP.

^c^ *P* values are from bivariate generalized estimating equation regressions clustered at the shopper level to account for repeated measurements.

Intraclass correlation statistics showed that 92.5% of the variation in spending on fruits, vegetables, nuts, and legumes was explained at the level of the shopper and 5.0% was explained at the level of the store. Table 3 shows differences in outcomes between SuperSNAP participants and shoppers in the comparison group during the period after the index date. The total purchases increased by $30.34 (95% CI, $26.81-$33.88; *P* < .001) in the SuperSNAP group compared with the group that never used the program.

**Table 3.** Changes in Purchases Associated With SuperSNAP Participation

Variable	Estimate (95% CI)^a^
All grocery store spending, $	30.34 (26.81 to 33.88)
Food and beverage spending, $	28.64 (25.87 to 31.41)
Fruits, vegetables, nuts, and legumes spending, $	31.84 (31.27 to 32.42)
Fruits, vegetables, nuts, and legumes spending, oz	294.52 (288.84 to 300.20)
Fruits, vegetables, nuts, and legumes spending among total food and beverage spending, %	14.52 (14.31 to 14.74)
Out-of-pocket fruits, vegetables, nuts, and legumes spending among total food and beverage spending, %	−20.14 (−20.61 to −19.67)
Spending on less healthy food categories, $	1.60 (0.67 to 2.53)
Spending on less healthy food categories, oz	22.95 (17.79 to 28.11)
Spending on less healthy food categories among total food and beverage spending, %	−4.51 (−4.74 to −4.27)
Out-of-pocket spending on less healthy food categories among total spending on less healthy food categories, %	−6.29 (−6.76 to −5.82)
Sugar-sweetened beverage spending, $	−1.83 (−2.36 to −1.30)
Sugar-sweetened beverage spending, oz	−40.22 (−54.52 to −25.92)
Sugar-sweetened beverage spending among total food and beverage spending, %	−3.75 (−3.97 to −3.53)
Out-of-pocket, sugar-sweetened beverage spending among total sugar-sweetened beverage spending, %	−4.39 (−4.93 to −3.86)

Abbreviation: SNAP, Supplemental Nutrition Assistance Program.

^a^ Estimates presented are from linear mixed models with terms for SuperSNAP (1 or 0), time (before SuperSNAP, 0; during SuperSNAP, 1), and a SuperSNAP × time product term. Models were also adjusted for month and year and duration of follow-up, with shopper-month as the unit of analysis. Models included 2 random-effects terms: shopper and shopper’s most used store for a given month. *P* < .001 for all estimates.

When categories of purchases were examined, spending on fruits, vegetables, nuts, and legumes increased by $31.84 (95% CI, $31.27-$32.42; *P* < .001) in the SuperSNAP group (complete model results are given in eTable 2 in the Supplement). The proportion of total food and beverage spending on fruits, vegetables, nuts, and legumes increased by 14.52% (95% CI, 14.31%-14.74%; *P* < .001) in the SuperSNAP group. This amounted to a monthly increase of 294.52 oz (95% CI, 288.84-300.20 oz; *P* < .001). In a mean household size of 2.24 individuals in the analytic sample, this represented 4.4 oz, or approximately 1 serving per person per day.^11^

Spending on less healthy food categories increased minimally in the SuperSNAP group compared with the group that never used the program (difference, $1.60; 95% CI, $0.67-$2.53; *P* < .001). As total spending increased, proportion of total food and beverage spending on less healthy food decreased by 4.51% (95% CI, 4.27%-4.74%; *P* < .001). SuperSNAP participation was not associated with increased out-of-pocket spending (ie, using non-SNAP benefits) on these items; out-of-pocket spending on less healthy items decreased by 6.29% (95% CI, 5.82%-6.76%; *P* < .001) in the SuperSNAP group compared with the group that did not use the program.

In the analysis of sugar-sweetened beverages, total spending on food and beverages decreased by $1.83 (95% CI, $1.30-$2.36; *P* < .001) and spending as a proportion of total spending on food and beverages decreased by 3.75% (95% CI, 3.53%-3.97%; *P* < .001). Out-of-pocket spending on food and beverages decreased by 4.39% (95% CI, 3.86%-4.93%; *P* < .001).

Results of sensitivity analyses in which the entire sample was examined were similar to results of the main analyses (eTable 3 in the Supplement). In the sensitivity analyses, spending on fruits, vegetables, nuts, and legumes was greater among SuperSNAP participants compared with those who never used the program (difference, $28.75; 95% CI, $25.95-$31.54; *P* < .001). Spending on less healthy foods was not substantially different, with a mean difference of −$1.66 (95% CI, −$7.27 to $3.95; *P* = .56). Spending on sugar-sweetened beverages was estimated to have decreased by $4.74 (95% CI, $1.75-$7.74; *P* = .002) or 4.5% (95% CI, 3.1%-5.9%; *P* < .001).

## Discussion

In this longitudinal cohort study, participation in SuperSNAP was associated with increased purchasing of fruits and vegetables. Propensity score–based analyses that incorporated data before and after SuperSNAP enrollment revealed that spending on fruits and vegetables was approximately $30 per month greater after SuperSNAP enrollment, with no meaningful increase in spending on less healthy foods and decreased spending on sugar-sweetened beverages. Sensitivity analyses that included the entire sample of SuperSNAP users showed similar findings.

This study makes 3 notable contributions to the literature on fruit and vegetable subsidies. First, prior studies^24,26,36,37,38,39^ have found that incentives may be associated with meaningful increases in purchasing of fruits and vegetables, but these studies typically examined programs based in smaller local food stores and farmers’ markets or that used paper-based enrollment and vouchers. The present study used data from a large supermarket chain and a streamlined online enrollment process and electronic issuance, all of which increase the potential for high-volume scalability. Second, in the present study, increases in healthy food purchasing occurred in a setting with a variety of less healthy and healthy food options, suggesting that programs such as SuperSNAP may be associated with improved health behaviors. Third, we found no meaningful increases in unhealthy food purchases (and decreased spending on sugar-sweetened beverages). Enrollment in SuperSNAP through clinics may have encouraged mental accounting of the $40 incentive, helping to restrict changes to purchases of healthier foods.^40,41^ Alternatively, if demand for healthier food among participants were greater than baseline resources, additional resources might be preferentially directed to meet this demand.

The findings of this study suggest directions for future work. Examination of how food incentives and subsidies may change diet quality, health outcomes, health care utilization, and health care cost is needed. Policies, such as the Gus Schumacher Nutrition Incentive Program, may play a key role in this research. Previous programs^42^ did not have a great emphasis on evaluation of potential health benefits, but examining whether these subsidy programs are associated with health benefits will be important ongoing work. Other future directions of research include examining potential program benefits for all members of the household (not just the enrolled individual, because food is shared within households), establishing the optimal amount and duration of these benefits, and examining possible heterogeneous treatment effects by social circumstances and clinical characteristics. In addition, how associations found in the study may apply to other samples and to SNAP beneficiaries overall should be evaluated.

### Strengths and Limitations

This study has strengths. This study used objective food purchasing data and compared SuperSNAP participants with a large number of SNAP beneficiaries throughout North Carolina. The consistency of results between 2 different analytic approaches, one of which emphasized internal validity and the other generalizability, suggests that the results observed in this study are likely to reflect the experience of SuperSNAP participants more broadly.

This study also has limitations. We had access only to shopper ID–level purchasing data; thus, we were not able to assess how changes in purchasing translated to changes in diet quality for individuals (if they did at all). However, we believe that a change in purchasing is a first step in the pathway to change in diet quality and improved health. Second, follow-up was relatively brief, and whether changes observed were sustained is not known. Third, how the results might have been affected by food purchases made outside the participating supermarket chain is unclear. Because we only had transaction data and not demographic, social, or clinical data, the possibility of unmeasured confounding cannot be ruled out. However, to confound the association between SuperSNAP participation and food purchases, the unmeasured factors would have had to affect food purchasing. Therefore, the preintervention data used for the propensity score analysis should help account for unmeasured factors because the associations, if any, of time-invariant demographic, social, and clinical characteristics with food purchasing would be reflected in food purchases before the index date. Furthermore, SuperSNAP participants were enrolled by clinicians for the purpose of receiving a benefit intended to improve diet-related chronic disease. Thus, the participants likely represent a unique subset of the overall population of SNAP beneficiaries who may be most likely to benefit from a program such as SuperSNAP. Whether the results observed in the group selected for SuperSNAP participation could be generalized to the broader population of SNAP beneficiaries is unclear. Reasons the results may not be generalizable include the characteristics of those enrolled and the setting of clinic-based enrollment, which may have worked synergistically with the cash value of the benefit provided to influence purchases of healthy food. Additional studies should explore these issues, potentially by using cash-benchmarked study designs that can isolate the impact of clinic-based approaches from the cash value of the benefit offered.^43^

## Conclusions

In this cohort study, SuperSNAP, which provides a fruit and vegetable subsidy to SNAP beneficiaries, was associated with increased purchasing of fruits and vegetables, a minimal increase in purchasing of less healthy foods, and decreased purchasing of sugar-sweetened beverages. Future studies should examine the potential effects of subsidies for the purchase of fruits and vegetable on diet quality, food security, health, and health care utilization. Given the high prevalence of food insecurity and its association with poor health outcomes,^44^ fruit and vegetable subsidies may be associated with important public health benefits in the US.

## Notes

Supplement. **eAppendix.** Technical Appendix **eTable 1.** Food and Beverage Categories and Their Rationale **eTable 2.** Full Regression Model for Amount Spent on Fruits, Vegetables, Nuts, and Legumes in Weighted Sample **eTable 3.** Sensitivity Analyses of Changes in Purchases Associated with SuperSNAP Participation, without Restricting to Those with Pre-Index Observation Time
eReferences

## References

[1] Mokdad AH, Ballestros K, Echko M, ; US Burden of Disease Collaborators. The state of US health, 1990-2016: burden of diseases, injuries, and risk factors among US states. JAMA. 2018;319(14):1444-1472. doi:10.1001/jama.2018.0158 29634829 PMC5933332

[2] Sotos-Prieto M, Bhupathiraju SN, Mattei J, . Association of changes in diet quality with total and cause-specific mortality. N Engl J Med. 2017;377(2):143-153. doi:10.1056/NEJMoa1613502 28700845 PMC5589446

[3] Sotos-Prieto M, Bhupathiraju SN, Mattei J, . Changes in diet quality scores and risk of cardiovascular disease among US men and women. Circulation. 2015;132(23):2212-2219. doi:10.1161/CIRCULATIONAHA.115.017158 26644246 PMC4673892

[4] Sugiyama T, Shapiro MF. The growing socioeconomic disparity in dietary quality: mind the gap. JAMA Intern Med. 2014;174(10):1595-1596. doi:10.1001/jamainternmed.2014.3048 25178585

[5] Wang DD, Leung CW, Li Y, . Trends in dietary quality among adults in the United States, 1999 through 2010. JAMA Intern Med. 2014;174(10):1587-1595. doi:10.1001/jamainternmed.2014.3422 25179639 PMC5924699

[6] Wang PY, Fang JC, Gao ZH, Zhang C, Xie SY. Higher intake of fruits, vegetables or their fiber reduces the risk of type 2 diabetes: a meta-analysis. J Diabetes Investig. 2016;7(1):56-69. doi:10.1111/jdi.12376 26816602 PMC4718092

[7] Alissa EM, Ferns GA. Dietary fruits and vegetables and cardiovascular diseases risk. Crit Rev Food Sci Nutr. 2017;57(9):1950-1962. doi:10.1080/10408398.2015.1040487 26192884

[8] Lelong H, Blacher J, Baudry J, . Individual and combined effects of dietary factors on risk of incident hypertension: prospective analysis from the NutriNet-Santé cohort. Hypertension. 2017;70(4):712-720. doi:10.1161/HYPERTENSIONAHA.117.09622 28760943

[9] Micha R, Shulkin ML, Peñalvo JL, . Etiologic effects and optimal intakes of foods and nutrients for risk of cardiovascular diseases and diabetes: systematic reviews and meta-analyses from the Nutrition and Chronic Diseases Expert Group (NutriCoDE). PLoS One. 2017;12(4):e0175149. doi:10.1371/journal.pone.0175149 28448503 PMC5407851

[10] He FJ, Nowson CA, Lucas M, MacGregor GA. Increased consumption of fruit and vegetables is related to a reduced risk of coronary heart disease: meta-analysis of cohort studies. J Hum Hypertens. 2007;21(9):717-728. doi:10.1038/sj.jhh.1002212 17443205

[11] Dietary Guidelines Advisory Committee. Scientific report of the 2020 Dietary Guidelines Advisory Committee: advisory report to the Secretary of Agriculture and the Secretary of Health and Human Services. US Dept of Agriculture, Economic Research Service; 2020. Accessed July 6, 2021. https://www.dietaryguidelines.gov/sites/default/files/2020-07/ScientificReport_of_the_2020DietaryGuidelinesAdvisoryCommittee_first-print.pdf

[12] Poti JM, Mendez MA, Ng SW, Popkin BM. Is the degree of food processing and convenience linked with the nutritional quality of foods purchased by US households? Am J Clin Nutr. 2015;101(6):1251-1262. doi:10.3945/ajcn.114.100925 25948666 PMC4441809

[13] Malik VS, Popkin BM, Bray GA, Després JP, Hu FB. Sugar-sweetened beverages, obesity, type 2 diabetes mellitus, and cardiovascular disease risk. Circulation. 2010;121(11):1356-1364. doi:10.1161/CIRCULATIONAHA.109.876185 20308626 PMC2862465

[14] Coleman-Jensen A, Rabbitt MP, Gregory CA, Singh A. Household food security in the United States in 2019. Accessed November 3, 2020. https://www.ers.usda.gov/publications/pub-details/?pubid=99281

[15] Feeding America. The impact of the coronavirus on local food insecurity. Accessed May 22, 2020. https://www.feedingamerica.org/sites/default/files/2020-05/Brief_Local%20Impact_5.19.2020.pdf

[16] Morales ME, Berkowitz SA. The relationship between food insecurity, dietary patterns, and obesity. Curr Nutr Rep. 2016;5(1):54-60. doi:10.1007/s13668-016-0153-y 29955440 PMC6019322

[17] Seligman HK, Schillinger D. Hunger and socioeconomic disparities in chronic disease. N Engl J Med. 2010;363(1):6-9. doi:10.1056/NEJMp1000072 20592297

[18] Seligman HK, Berkowitz SA. Aligning programs and policies to support food security and public health goals in the United States. Annu Rev Public Health. 2019;40:319-337. doi:10.1146/annurev-publhealth-040218-044132 30444684 PMC6784838

[19] Food and Nutrition Service, US Department of Agriculture. Supplemental Nutrition Assistance Program (SNAP). Accessed February 19, 2020. https://www.fns.usda.gov/snap/supplemental-nutrition-assistance-program

[20] Canning P, Stacy B. The Supplemental Nutrition Assistance Program (SNAP) and the economy: new estimates of the SNAP multiplier. Accessed July 3, 2020. https://www.ers.usda.gov/publications/pub-details/?pubid=93528

[21] Gundersen C. Food insecurity is an ongoing national concern. Adv Nutr. 2013;4(1):36-41. doi:10.3945/an.112.003244 23319121 PMC3648737

[22] Carlson S. More adequate SNAP benefits would help millions of participants better afford food. Center on Budget and Policy Priorities. Accessed July 3, 2020. https://www.cbpp.org/research/food-assistance/more-adequate-snap-benefits-would-help-millions-of-participants-better

[23] Mozaffarian D, Liu J, Sy S, . Cost-effectiveness of financial incentives and disincentives for improving food purchases and health through the US Supplemental Nutrition Assistance Program (SNAP): a microsimulation study. PLoS Med. 2018;15(10):e1002661. doi:10.1371/journal.pmed.1002661 30278053 PMC6168180

[24] Olsho LE, Klerman JA, Wilde PE, Bartlett S. Financial incentives increase fruit and vegetable intake among Supplemental Nutrition Assistance Program participants: a randomized controlled trial of the USDA Healthy Incentives Pilot. Am J Clin Nutr. 2016;104(2):423-435. doi:10.3945/ajcn.115.129320 27334234

[25] Berkowitz SA, O’Neill J, Sayer E, . Health center–based community-supported agriculture: an RCT. *Am J Prev Med*. 2019;57(6 suppl 1):S55-S64. doi:10.1016/j.amepre.2019.07.015 PMC6874748 31522922

[26] Yoder AD, Proaño GV, Handu D. Retail nutrition programs and outcomes: an evidence analysis center scoping review. J Acad Nutr Diet. Published online November 20, 2020. doi:10.1016/j.jand.2020.08.080 33229206

[27] Reinvestment Partners. SuperSNAP. Accessed November 4, 2020. https://reinvestmentpartners.org/what-we-do/produce-prescriptions/supersnap-1.html

[28] National Institute of Food and Agriculture, US Department of Agriculture. Gus Schumacher Nutrition Incentive Program. Accessed November 3, 2020. https://nifa.usda.gov/program/gus-schumacher-nutrition-incentive-grant-program

[29] Healthy Food America. Healthy food pricing incentives: a systematic review of current evidence. Accessed November 4, 2020. https://www.healthyfoodamerica.org/healthy_food_pricing_incentives_a_systematic_review_of_current_evidence

[30] Hager ER, Quigg AM, Black MM, . Development and validity of a 2-item screen to identify families at risk for food insecurity. Pediatrics. 2010;126(1):e26-e32. doi:10.1542/peds.2009-3146 20595453

[31] Lacko A, Ng SW, Popkin B. Urban vs. rural socioeconomic differences in the nutritional quality of household packaged food purchases by store type. Int J Environ Res Public Health. 2020;17(20):7637. doi:10.3390/ijerph17207637 33092077 PMC7589700

[32] Ng SW, Hollingsworth BA, Busey EA, Wandell JL, Miles DR, Poti JM. Federal nutrition program revisions impact low-income households’ food purchases. Am J Prev Med. 2018;54(3):403-412. doi:10.1016/j.amepre.2017.12.003 29455757 PMC5820777

[33] Berkowitz SA, Delahanty LM, Terranova J, . Medically tailored meal delivery for diabetes patients with food insecurity: a randomized cross-over trial. J Gen Intern Med. 2019;34(3):396-404. doi:10.1007/s11606-018-4716-z 30421335 PMC6420590

[34] Thomas LE, Li F, Pencina MJ. Overlap weighting: a propensity score method that mimics attributes of a randomized clinical trial. JAMA. 2020;323(23):2417-2418. doi:10.1001/jama.2020.7819 32369102

[35] Li F, Morgan KL, Zaslavsky AM. Balancing covariates via propensity score weighting. J Am Stat Assoc. 2018;113(521):390-400. doi:10.1080/01621459.2016.126046629930437

[36] Gittelsohn J, Trude ACB, Kim H. Pricing strategies to encourage availability, purchase, and consumption of healthy foods and beverages: a systematic review. Prev Chronic Dis. 2017;14:E107. doi:10.5888/pcd14.170213 29101767 PMC5672888

[37] Engel K, Ruder EH. Fruit and vegetable incentive programs for Supplemental Nutrition Assistance Program (SNAP) participants: a scoping review of program structure. Nutrients. 2020;12(6):1676. doi:10.3390/nu12061676 32512758 PMC7352438

[38] Moran A, Thorndike A, Franckle R, . Financial incentives increase purchases of fruit and vegetables among lower-income households with children. Health Aff (Millwood). 2019;38(9):1557-1566. doi:10.1377/hlthaff.2018.05420 31479362

[39] Seguin RA, Morgan EH, Hanson KL, . Farm Fresh Foods for Healthy Kids (F3HK): an innovative community supported agriculture intervention to prevent childhood obesity in low-income families and strengthen local agricultural economies. BMC Public Health. 2017;17(1):306. doi:10.1186/s12889-017-4202-2 28390403 PMC5385092

[40] Mancino L, Guthrie J, Just DR. Overview: exploring ways to encourage healthier food purchases by low-income consumers—lessons from behavioral economics and marketing. Food Policy. 2018;79:297-299. doi:10.1016/j.foodpol.2018.03.007

[41] Audet AJ, Zezza MA. How behavioral economics can advance the design of effective clinician incentive programs. *Health Affairs* blog. Accessed December 4, 2020. https://www.healthaffairs.org/do/10.1377/hblog20150915.050556/full/

[42] Food and Nutrition Service, US Department of Agriculture. FINI Grant Program. Accessed July 6, 2021. https://www.fns.usda.gov/snap/FINI-Grant-Program

[43] Berkowitz SA, Edwards ST, Polsky D. Cash benchmarking for integrated health care and human services interventions: finding the value added. Health Aff (Millwood). 2020;39(4):582-586. doi:10.1377/hlthaff.2019.01579 32250693 PMC7724638

[44] Gundersen C, Ziliak JP. Food insecurity and health outcomes. Health Aff (Millwood). 2015;34(11):1830-1839. doi:10.1377/hlthaff.2015.0645 26526240

