# Error in Food Purchasing Data in Study of Fruit and Vegetable Subsidy Program Among US Individuals With Low Income

**DOI:** 10.1001/jamanetworkopen.2021.42888

**Published:** 2021-12-13

In the Original Investigation titled “Association of a Fruit and Vegetable Subsidy Program With Food Purchases by Individuals With Low Income in the US,”^1^ published on August 11, 2021, an error in the food purchasing data the authors received from the supermarket chain resulted in errors in the published article. The corresponding author has explained the errors in a Comment.^2^ The errors apply proportionally to both the intervention group and the comparison group. In some instances, when individuals used more than 1 payment method in the same transaction (eg, both cash and credit card), called a “split-tender” transaction, there was duplication of the purchase record. The error affects descriptive statistics and point estimates and 95% CIs of analyses (eg, the primary outcome is that the intervention group spent approximately $24 dollars more per month on fruits and vegetables compared with $30 as previously reported). Also, the number of comparison group participants increased from 33 246 to 42 716, related to cohort construction, which removed outlier shopper identification values at a cutoff level corresponding to the top 1% of purchases. In the corrected dataset, this level changed from $1452 to $1008, and the distribution of expenditures changed, resulting in more included comparison group participants. Finally, 3 outcomes related to out-of-pocket expenditures (ie, expenditures that were not related to food supplement programs) were removed. The authors have confirmed that corrections to address these errors do not change the direction and statistical significance of study outcomes, interpretations, or conclusions reported. To address these errors, corrections have been made to the Abstract, Key Points, Methods and Results sections, Tables 2 and 3, and the Supplement. This article has been corrected.^1^
