# Supplementary material for: Association of a Fruit and Vegetable Subsidy Program With Food Purchases by Individuals With Low Income in the US
Source: JAMA Netw Open. 2021 Aug 11;4(8):e2120377. doi: 10.1001/jamanetworkopen.2021.20377 (PMC8358732; doi:10.1001/jamanetworkopen.2021.20377)
Supplement: Supplement. — eAppendix. Technical Appendix eTable 1. Food and Beverage Categories and Their Rationale eTable 2. Full Regression Model for Amount Spent on Fruits, Vegetables, Nuts, and Legumes in Weighted Sample eTable 3. Sensitivity Analyses of Changes in Purchases Associated with SuperSNAP Participation, without Restricting to Those with Pre-Index Observation Time eReferences [file jamanetwopen-e2120377-s001.pdf]

Supplemental Online Content

Berkowitz SA, Curran N, Hoeffler S, Henderson R, Price A, Ng SW. 'Association of a fruit and vegetable subsidy program with food purchases by individuals with low income in the US. *JAMA Netw Open.* 2021;4(8):e2120377. doi:10.1001/jamanetworkopen.2021.20377

eAppendix. Technical Appendix

eTable 1. Food and Beverage Categories and Their Rationale

eTable 2. Full Regression Model for Amount Spent on Fruits, Vegetables, Nuts, and Legumes in Weighted Sample

eTable 3. Sensitivity Analyses of Changes in Purchases Associated with SuperSNAP Participation, without Restricting to Those with Pre-Index Observation Time

eReferences

This supplemental material has been provided by the authors to give readers additional information about their work.

© 2021 Berkowitz SA et al. *JAMA Network Open.*

Technical Appendix

Data pre-processing

To account for outliers, shopper ID numbers in the top 1% of monthly food and beverage spending (>$1452/month) were excluded as investigation of the transaction records suggested that these were commonly used by multiple households (e.g., a cashier may scan one of these shopper ID numbers to provide discounts to those who forgot their shopper ID card).

List of variables for propensity score estimation

The following variables were used for propensity score estimation: total expenditure, food and beverage expenditure, chocolate, candy, and gum expenditure, percentage of transactions that used a coupon, dessert and sweet snacks expenditure, fruit, vegetable, legumes and nuts expenditure, percentage of total expenditures for fruit, vegetables, nuts, and legumes, volume of fruit, vegetables, nuts, and legumes (in ounces) purchased, junk food expenditure, volume of junk food purchased (in ounces), sugar sweetened beverage expenditure, volume of sugar sweetened beverage expenditure (in ounces), expenditure on WIC-eligible fruit, vegetables, nuts, and legumes, percentage of food and beverage purchases paid for out of pocket (e.g., not by SNAP funds), percentage of chocolate, candy, and gum paid for out of pocket, percentage of dessert and sweet snacks paid for out of pocket, percentage of fruit, vegetables, nuts, and legumes paid for out of pocket, percentage of junk food paid for out of pocket, percentage of processed meats and seafood paid for out of pocket, percentage of sugar sweetened beverages paid for out of pocket, percentage of sweeteners and toppings paid for out of pocket, percentage of salty snacks paid for out of

© 2021 Berkowitz SA et al. *JAMA Network Open.*

pocket, expenditures on processed meats and seafood, percentage of food and beverage spending that came from SNAP benefits, percentage of chocolate, candy, and gum spending from SNAP benefits, percentage of dessert and sweet snacks spending from SNAP benefits, percentage of fruit, vegetables, nuts, and legumes spending from SNAP, percentage of fruit, junk food spending from SNAP, percentage of processed meat and seafood spending from SNAP, percentage of SSB spending from SNAP, percentage of sweeteners and toppings from SNAP, expenditures on sweeteners and toppings, expenditures on salty snacks, percentage of food and beverage expenditure spent on sugar sweetened beverage, index month, and amount of pre-index follow-up time.

© 2021 Berkowitz SA et al. *JAMA Network Open.*

eTable 1: Food and Beverage Categories and their Rationale	
Food purchase measures, all monthly	Rationale and link to health outcomes
Foods covered by SuperSNAP fruit and vegetable incentive	• Important sources of vitamins and fiber<br>• High consumption associated with lower cardiovascular disease risk.[1]<br>• Underconsumed in the US[2]<br>• WIC approved items
Fruit, vegetables, nuts and legumes	• Important sources of vitamins and fiber<br>• High consumption associated with lower cardiovascular disease risk.[1]<br>• Underconsumed in the US[2]
Sugar-sweetened beverages (SSBs) and less healthy foods (processed meats, desserts, sweet snacks, salty snacks, sweeteners and toppings, candy and chocolate)	• Large contributors of total energy, sugar, saturated fat and sodium in US diet[3]<br>• Overconsumption of processed meats, desserts, sweet snacks, salty snacks, sweeteners and toppings, candy and chocolate in the US[2]<br>• SSBs independently linked to obesity and diabetes[4]

© 2021 Berkowitz SA et al. *JAMA Network Open.*

**eTable 2: Full Regression model for Amount Spent on Fruits, Vegetables, Nuts, and Legumes in weighted sample**					
Fixed Effects Parameters					
	Estimate	Standard Error	P	Lower 95% CI	Upper 95% CI
Intercept	40.52	0.43	<.001	39.68	41.36
Participated in SuperSNAP (Reference = did not participate)	-2.85	1.20	0.018	-5.19	-0.50
Time (before or after index date)	-7.50	0.28	<.001	-8.04	-6.96
Time*Participated in SuperSNAP = 1 (reference: Time* Participated in SuperSNAP = 0)	31.84	0.29	<.001	31.27	32.42
Month and Year of Observation					
October 2019	-16.66	0.35	<.001	-17.35	-15.97
November 2019	-14.63	0.33	<.001	-15.27	-14.00
December 2019	-15.96	0.31	<.001	-16.57	-15.34
January 2020	-13.92	0.29	<.001	-14.48	-13.36
February 2020	-10.86	0.26	<.001	-11.37	-10.36
March 2020	-2.53	0.25	<.001	-3.01	-2.04
April 2020 (Reference)	0.00	n/a	n/a	n/a	n/a
Follow-up time, months	3.09	0.10	<.001	2.89	3.29
Covariance Parameters from Random Effects Portion of Model					
	Estimate	Standard Error	P		
Store random intercept	25.74	2.56	<.001		
Shopper ID random intercept	520.74	5.31	<.001		
Residual	11.21	0.04	<.001		

Results from Linear Mixed model including the above terms, and with a random intercept term for shopper ID and a random intercept term for the store at which each shopper ID number was used most often in a given month. Model includes overlap weights. Time indicates whether the observation occurred before or after the index date, which is the date of SuperSNAP enrollment for those who participated in SuperSNAP and a randomly chosen date for those who did not participate in SuperSNAP. The Time*Participated in SuperSNAP product term indicates whether the change in amount spent is greater for those who participated in SuperSNAP, after their index date, than those who did not participate.
CI = Confidence Interval
n/a = not applicable

© 2021 Berkowitz SA et al. *JAMA Network Open.*

eTable 3: Sensitivity Analyses of Changes in Purchases Associated with SuperSNAP Participation, without Restricting to Those with Pre-Index Observation Time

Variable	Estimate	Lower 95% CI	Upper 95% CI	P
All Grocery Store Spending, $	17.82	-3.91	39.55	0.11
Food and Beverage Spending, $	18.71	1.98	35.44	0.03
Fruits, Vegetables, Nuts, and Legume Spending, $	28.75	25.95	31.54	<.001
Fruits, Vegetables, Nuts, and Legume Spending, oz	263.33	236.25	290.41	<.001
Fruits, Vegetables, Nuts, and Legume Spending as Share of Total Food and Beverage Spending	16.18%	15.21%	17.16%	<.001
Out of Pocket Fruits, Vegetables, Nuts, and Legume Spending as Share of Total Food and Beverage Spending	-19.91%	-22.76%	-17.06%	<.001
Spending on Less Healthy Food Categories, $	-1.66	-7.27	3.95	0.56
Spending on Less Healthy Food Categories, oz	-12.27	41.16	16.61	0.41
Spending on Less Healthy Food Categories as Share of Total Food and Beverage Spending	-5.20%	-6.56%	-3.84%	<.001
Out of Pocket Spending on Less Healthy Food Categories as Share of Total Spending on Less Healthy Food Categories	-4.00%	-6.61%	-1.39%	0.003
Sugar Sweetened Beverage Spending, $	-4.74	-7.74	-1.75	0.002
Sugar Sweetened Beverage Spending, oz	-156.35	-234.72	-77.99	<.001
Sugar Sweetened Beverage Spending, as Share of Total Food and Beverage Spending	-4.51%	-5.89%	-3.12%	<.001
Out of Pocket Sugar Sweetened Beverage Spending, as Share of Total Sugar Sweetened Beverage Spending	-4.33%	-7.37%	-1.30%	0.005

Estimates presented are from linear mixed models with terms for SuperSNAP (1/0), time (before SuperSNAP = 0, during = 1) and a SuperSNAP-by-time product term. Models also adjusted for month and year, and duration of follow-up, with the shopper-month as the unit of analysis. Models include two random effects terms: shopper and shopper's most used store for a given month.

© 2021 Berkowitz SA et al. *JAMA Network Open.*

Appendix References

1. He FJ, Nowson CA, Lucas M, MacGregor GA. Increased consumption of fruit and vegetables is related to a reduced risk of coronary heart disease: meta-analysis of cohort studies. *J Hum Hypertens*. 2007;21(9):717-728. doi:10.1038/sj.jhh.1002212

2. Dietary Guidelines Advisory Committee. *Scientific Report of the 2020 Dietary Guidelines Advisory Committee: Advisory Report to the Secretary of Agriculture and the Secretary of Health and Human Services.* U.S. Department of Agriculture, Economic Research Service; 2020.

3. Poti JM, Mendez MA, Ng SW, Popkin BM. Is the degree of food processing and convenience linked with the nutritional quality of foods purchased by US households? *Am J Clin Nutr*. 2015;101(6):1251-1262. doi:10.3945/ajcn.114.100925

4. Malik VS, Popkin BM, Bray GA, Després J-P, Hu FB. Sugar Sweetened Beverages, Obesity, Type 2 Diabetes and Cardiovascular Disease risk. *Circulation*. 2010;121(11):1356-1364. doi:10.1161/CIRCULATIONAHA.109.876185

© 2021 Berkowitz SA et al. *JAMA Network Open.*
